# Attitudes and behaviors of Japanese physicians concerning withholding and withdrawal of life-sustaining treatment for end-of-life patients: results from an Internet survey

**DOI:** 10.1186/1472-6939-8-7

**Published:** 2007-06-19

**Authors:** Seiji Bito, Atsushi Asai

**Affiliations:** 1 National Hospital Organization Tokyo Medical Center, 2-5-1, Higashigaoka, Meguro-ku, Tokyo 152-8902, Japan; 2 Kumamoto University, 1-1-1, Honjo, Kumamoto, Kumamoto, 860-8556, Japan

## Abstract

**Background:**

Evidence concerning how Japanese physicians think and behave in specific clinical situations that involve withholding or withdrawal of medical interventions for end-of-life or frail elderly patients is yet insufficient.

**Methods:**

To analyze decisions and actions concerning the withholding/withdrawal of life-support care by Japanese physicians, we conducted cross-sectional web-based internet survey presenting three scenarios involving an elderly comatose patient following a severe stroke. Volunteer physicians were recruited for the survey through mailing lists and medical journals. The respondents answered questions concerning attitudes and behaviors regarding decision-making for the withholding/withdrawal of life-support care, namely, the initiation/withdrawal of tube feeding and respirator attachment.

**Results:**

Of the 304 responses analyzed, a majority felt that tube feeding should be initiated in these scenarios. Only 18% felt that a respirator should be attached when the patient had severe pneumonia and respiratory failure. Over half the respondents felt that tube feeding should not be withdrawn when the coma extended beyond 6 months. Only 11% responded that they actually withdrew tube feeding. Half the respondents perceived tube feeding in such a patient as a "life-sustaining treatment," whereas the other half disagreed. Physicians seeking clinical ethics consultation supported the withdrawal of tube feeding (OR, 6.4; 95% CI, 2.5–16.3; P < 0.001).

**Conclusion:**

Physicians tend to harbor greater negative attitudes toward the withdrawal of life-support care than its withholding. On the other hand, they favor withholding invasive life-sustaining treatments such as the attachment of a respirator over less invasive and long-term treatments such as tube feeding. Discrepancies were demonstrated between attitudes and actual behaviors. Physicians may need systematic support for appropriate decision-making for end-of-life care.

## Background

As medical technology becomes more advanced, judgments about whether to undertake invasive medical procedures have increasingly become a serious and difficult issue to resolve; this is true not only for patients in whom medical testing clearly demonstrates an end-of-life status but also for patients in a comatose state with very little prospect of recovery and for frail, elderly individuals [1-3]. To resolve these issues, various points must be clarified. For example, the fact that terms such as "end-of-life," "death with dignity," and "life-sustaining treatment" that are generally used as if their definitions were unequivocal are, in reality, extremely relative in nature and entail significant differences in nuance depending on the circumstances and the manner in which individuals understand these terms [4,5]. In reality, it is very difficult to assign a definition to a term that portrays an image of "life-sustaining treatment." There is further debate as to whether joint or individual consideration is appropriate for issues such as candidate suitability for various medical interventions and withholding or withdrawal of medical interventions [6].

Some studies have indicated the effect of cultural factors and attitudes toward decision-making in end-of-life care [7-9]. We might expect discrepancies in public awareness with respect to these topics; however, there are also questions regarding the degree of the differences in the awareness of the aforementioned topics within a particular group of physicians. Although some surveys have investigated physicians' attitudes toward end-of-life care and life-sustaining treatment [8-11], evidence concerning how Japanese physicians think and behave in specific clinical situations that involve withholding or withdrawal of medical interventions is yet insufficient.

Our research first entailed an anonymous web-based internet survey of physicians regarding general discrepancies in clinical and ethical judgment in the withholding or withdrawal of potentially life-extending medical interventions. We then made a comparative study of the relationship between the distribution of awareness, differences in the distribution of physician characteristics, and discrepancies in clinical judgment.

We also investigated the extent to which physicians utilize conferencing, clinical ethicists, and ethics committees in cases of difficult ethical judgments.

## Methods

We carried out a cross-sectional internet survey targeting physicians who self-accessed the survey homepage that was advertised through mailing lists, public medical journals. The survey did not involve a sampling process using means such as membership lists of specific medical organizations. For the survey, cooperation was sought from a non-specific pool of physicians. Further, it was anonymous in nature, and accessing the survey homepage was regarded as consent for survey participation. Encouragement toward accessing the homepage was limited to advertising through physician mailing lists, academic journals, and commercial medical journals. Four mailing lists were used for the advertisement: "Total Family Care" mailing list comprising approximately 2,500 primarily independent practitioners and primary care physicians, "Internist" mailing list comprising approximately 1,000 of the board members of the Japanese Society of Internal Medicine, "pEBM" mailing list comprising primarily evidence-based medicine (EBM) physicians, and "EML" mailing list comprising primarily emergency care providers. Journal advertisements were printed in bimonthly and biweekly Japanese medical journals in general medical fields with an emphasis on those for internal medicine.

The survey questions investigated the awareness regarding the withholding or withdrawal of potentially "life-extending treatment" in three case scenarios pertaining to medical intervention, namely, Case 1, Case 2, and Case 3. These three scenarios concerned judgment for the initiation/withholding of tube feeding for an elderly individual in a stroke-induced comatose state with a high potential for long-term prolongation, judgment for the attachment/withholding of a respirator in a patient with an identical status to the above patient with the additional occurrence of severe pneumonia, and judgment for the discontinuation/withdrawal of artificial feeding when a patient is in a prolonged comatose state for more than 6 months and the withdrawal of tube feeding has been requested by the patient's family (Appendix). Based on these three scenarios, the survey sought responses as to whether the available treatment options should or should not be withheld or withdrawn. The survey also used an analogous method to seek responses concerning actual actions in routine practice. Further, the survey also assessed whether physicians viewed two particular medical interventions as either "life-sustaining treatment" or not these two interventions were the continuation of artificial feeding through a gastrostomy for a patient in the third aforementioned scenario and the attachment of an artificial respirator when this patient developed severe pneumonia and would likely require more than 7 days until separation from the respirator could be undertaken.

In addition to the case scenario questions, we inquired the extent to which physicians make use of resources such as conferencing, consultation with clinical ethicists, and application to ethics committees when faced with difficult cases pertaining to ethical judgment.

Data input was carried out through an Internet homepage created specifically for the survey research, and electronic mail was not used. Physicians were asked to input their age, sex, number of years since graduation, and specialty; however, physicians did not provide any other personally identifying information. To achieve complete anonymity of personal information in the research, the server storing the response data was set up in a data center unaffiliated with the researchers. Information obtained by the researchers from the data center was completely anonymized, and researchers were entirely unable to obtain the internet protocol (IP) address of the respondents or other such information. For furthering the efforts to prevent the identification of individuals, the survey was carried out completely on a volunteer basis with no acknowledgements or incentives provided. The survey was opened on January 10, 2005 and remained open until March 31, 2005.

After all the survey mechanisms were complete, the researchers analyzed the anonymous data. To cleanse the data of the possibility of the same physician responding multiple times, data with identical answers for physician age, gender, field of practice, and employing institution and having a 75% or greater concordance in responses to the other questions were treated as responses from the same physician. In these cases, only data from the initial access were selected, and data from the second and subsequent accesses were deleted. In addition to descriptive statistics for each question, the statistical analysis included the calculation of kappa values for concordance between awareness and actual practice of withholding or withdrawal of specific treatments in each scenario and for concordance in responses across scenarios. The discrepancies in judgment-related awareness of the treatments were also compared by physician characteristics. The relationships between the attitudes with regard to the judgments in cases 1, 2, and 3 and the physician characteristics and experiences concerning ethical matters were analyzed using a logistic regression model. Odds ratios (OR) and their 95% confidence intervals (CI) were calculated.

The conduct of the research was approved by Tokyo Medical Center Ethics Committee in November, 2004.

## Results

Responses were received from 307 individuals. In this group, one response was deemed not to have been provided by a physician, and two couples of responses were regarded as duplicate responses; these were deleted, and the remaining 304 responses were analyzed. The sex and age distribution was as follows: female, 15%; male, 85%; 39 yrs or lower, 41%; 40–49 yrs, 42%; and 50 yrs or higher, 15%. The distribution of the fields of practice was as follows: pediatrics, 2%; family practice, 11%; general internal medicine, 44%; specialty of internal medicine, 21%; surgery-related, 7%; and emergency medicine, 9%. The distribution of the size of the employing facilities and that of the size of the employing facilities were shown in Table 1.

**Table 1:** Distributions of the respondents (N = 304)

Age stratum	(%)
39 years or younger	41
40–49 years	42
50 years or older	15
Missing data	2
Sex	
Female	15
male	85
Fields of practice	
Pediatrics	2
Family practice	11
General internal medicine	44
Specialty internal medicine	21
Surgery-related	7
Emergency medicine	9
The other fields	7
Size of employing facilities	
Office-based clinic	24
1–100 beds	13
101–250 beds	17
251–500 beds	17

501 or more beds	28

In response to the question "To what extent do you consult with your colleagues regarding the attachment or disconnection of an artificial respirator to patients, the initiation or withdrawal of tube feeding, and other such matters of judgment?," 50% of the physicians responded with "frequently," 42% answered "very seldom," and 8% responded with "no experience of consultation."

In response to the question "To what extent do you use in-hospital conferences or other such means to discuss the attachment or disconnection of an artificial respirator to patients, the initiation or withdrawal of tube feeding, and other such matters of judgment?," 28% of the physicians responded with "frequently," 45% answered "very seldom," and 27% responded with "no experience of consultation."

In response to the question "Have you ever held consultations with ethics committees, medical ethicists, or other such specialists regarding the attachment or disconnection of an artificial respirator to patients, the initiation or withdrawal of enteric nutrition administration, or other such matters of judgment?," 9% of the physicians responded with "have experience of consultation," whereas more than 90% of the physicians answered that they had no experience of consultation.

With regard to Case 1, the question "What do you think should be done with regard to the initiation of enteric feeding for the aforementioned patient?" was asked. 55% of the physicians responded with "enteric nutrition should be initiated either by nasogastric intubations or gastrostomy," 15% answered that "initiation of enteric nutrition should be withheld," and 30% responded with "judgment is not possible based only on the information above." In response to the question "What do you do with regard to the initiation of enteric nutrition for such a type of patient?," 70% of the physicians responded with "usually, initiate enteric nutrition," 11% answered "usually, withhold the initiation of enteric nutrition," and 14% responded with "cannot say one more than the other." The number of physicians who selected "have not encountered a situation such as the one above" was only 5% (Table 2).

**Table 2:** "Should/should not" awareness and what is actually done answers toward the Cases (n = 304)

	Should/should not" awareness of treatment options (%)			
	Should be done (withdrawn)	Should not be done (not withdrawn)	Judgment is not possible	
	
	What is actually being done (%)			
	Usually, initiate	Usually, withhold	Cannot say one more than the other	Have not encountered
Case1	55	15	30	
(Initiation of tube feeding)	70	11	14	5
Case2	18	54	28	
(Attachment of respirator)	18	59	19	4
Case3	16	53	31	
(Withdrawal of tube feeding)	11	53	16	20

Among the 166 physicians that responded with "enteric nutrition should be initiated either by nasogastric intubation or gastrostomy," 150 answered "usually, initiate enteric nutrition" (Kappa = 0.46, P < 0.001); whereas, among the 46 physicians that responded with "initiation of enteric nutrition should be withheld," 22 physicians, i.e., roughly half, answered "usually, withhold the initiation of enteric nutrition" (Kappa = 0.50, P < 0.001).

In response to Case 2, the question "What do you think should be done with regard to the attachment of an artificial respirator for such a type of patient?" was asked. While 18% of the physicians responded with "a respirator should be attached," a majority comprising 54% of the physicians answered "attachment of an artificial respirator should be withheld," and 28% responded with "judgment is not possible based only on the information above." In response to the question "What do you do with regard to the attachment of an artificial respirator for such a type of patient?," 18% of the physicians answered "usually, attach the respirator," 59% selected "usually, withhold the attachment of the respirator," 19% responded with "cannot say one more than the other," and 4% answered "have not encountered a situation such as that the one above" (Table 2).

Among the 163 physicians that responded with "attachment of an artificial respirator should be withheld," 140 selected "usually, withhold the attachment of an artificial respirator" (Kappa = 0.60, P < 0.001). Additionally, of the 166 physicians who responded in Case 1 that "Enteric nutrition should be initiated either by nasogastric intubation or gastrostomy," only 45 of these responded with "respirator should be attached" in Case 2, and 84 physicians answered "attachment of the respirator should be withheld." Further, of the 46 physicians who responded with "initiation of enteric nutrition should be withheld" in Case 1, 42 individuals, i.e., almost all, answered "attachment of an artificial respirator should be withheld" (Kappa = 0.22, P < 0.001).

For Case 3, the question asked was "In circumstances such as those described above, do you think that enteric nutrition should be withdrawn pursuant to a family request?" To this question, 16% of the physicians responded with "should be withdrawn," 53% answered "should not be withdrawn," and 31% selected "judgment is not possible based only on the information above." In response to the question "What do you do regarding the withdrawal of artificial nutrition in cases such as the one described above?," 11% of the physicians responded with "usually, withdraw," 53% answered "usually, do not withdraw," 16% selected "cannot say one more than the other," and 20% responded with "have not encountered a situation such as the one above" (Table 2).

**Figure 1:**
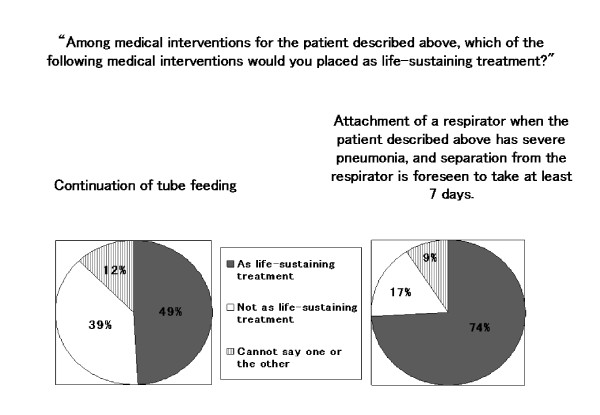
**Physicians' perceptions toward continuation of tube feeding and attachment of a respirator for seriously ill patients**. Toward the questions asking about continuation of tube feeding and about attachment of a respirator when a persistent coma elderly patient by stroke in the scenario has severe pneumonia, three of four physicians answered that attachment of a respirator would be placed as "life-sustaining treatment" while about a half of the physicians answered that continuation of tube feeding is placed as "life-sustaining treatment."

With regard to Case 3, physicians were also asked "Among medical interventions for the patient described above, which of the following medical interventions you would place in the category of life-sustaining treatment?" Physicians had to respond to two interventions: "The continuation of enteric nutrition by gastrostomy" and "The attachment of an artificial respirator when the patient described above has severe pneumonia, and separation from the respirator is foreseen to take at least 7 days." Three alternatives were available as responses: "in the life-sustaining treatment category," "not in the life-sustaining treatment category," and "cannot say one or the other." Regarding the former intervention, the proportion of the respondents selecting each alternative was 49%, 39%, and 12%, respectively, demonstrating a great disparity in opinions. Regarding the latter intervention, the proportion of respondents selecting each alternative was 74%, 17%, and 9%, respectively, with the large majority of physicians selecting "in life-sustaining treatment category" (Figure 1).

Further, with regard to the aforementioned patient, the physicians were also asked whether "the continuation of enteric feeding by gastrostomy" and "the connection to an artificial respirator when the above-described patient has severe pneumonia and recovery is foreseen to take at least 7 days" were regarded as "medical interventions futile for the patient." Regarding the former intervention, 28% of the physicians responded with "I believe that the intervention is futile for the patient," 44% answered "I do not believe that the intervention is futile," and 26% selected "cannot say either one." Regarding the latter intervention, the proportion of respondents selecting each alternative was 52%, 23%, and 25%, respectively.

Among the 47 physicians that responded with "should be withdrawn" to the aforementioned question regarding Case 3, namely, "In circumstances such as those described above, do you think that enteric nutrition should be withdrawn pursuant to a family request?," 22 physicians, i.e., less than half, responded with "usually, withdraw" (Kappa = 0.49, P < 0.001). Among the 160 physicians that responded with "should not be withdrawn," only 3 physicians answered "usually, withdraw."

With regard to the patient in Case 3, among the 149 physicians that responded that "the continuation of enteric nutrition by gastrostomy" was "in the life-sustaining treatment category," 71 physicians, i.e., less than half, answered that this medical intervention was "futile for the patient." Conversely, among the 86 physicians who answered that this medical intervention was "futile for the patient," a majority of 71 physicians responded that this medical intervention was "In the life-sustaining treatment category" (Kappa = 0.38, P < 0.001).

Among the 225 physicians who responded that "the connection to an artificial respirator when the above-described patient has severe pneumonia and recovery is foreseen to take at least 7 days" was "in the life-sustaining treatment category," 150 physicians answered that this medical intervention was "futile for the patient"; however, nearly all the physicians who answered that this medical intervention was "futile for the patient" also responded that this medical intervention was "in the life-sustaining treatment category" (Kappa = 0.44, P < 0.001).

Table 3 shows the relationship between responses indicating that a certain treatment "should be withheld" in Case 1 and Case 2 with factors such as physician characteristics, field of practice, employing facility characteristics, and experience in ward or conference consultation concerning artificial respirator attachment and indication of enteric nutrition. Table 3 also shows the relationships of these factors to the response that the continuation of enteric nutrition "should be withdrawn" in Case 3.

**Table 3:** Predictors of the attitudes for withholdings in Case 1 and 2, and for withdrawal of tube feeding in Case 3*

	**Odds Ratio [95% CI]**		
	
**Predictors**	**Case 1**	**Case 2**	**Case 3**
Female (male)	0.7 [0.3 – 1.5]	0.6 [0.4 –1.1]	1.2 [0.5 – 2.7]
Age over 50 (younger age)	0.8 [0.4 – 3.0]	0.7 [0.4 – 1.5]	1.2 [0.4 – 3.1]
Surgical specialty (others)	0.5 [0.2 – 2.1]	0.6 [0.3 – 1.2]	0.3 [0.1 – 1.1]
Large volume facility over 500 beds (under 500 beds)	0.3 [0.1 – 0.9]	1.3 [0.7 – 2.4]	1.3 [0.6 – 2.9]
Primary care clinic (hospital)	0.9 [0.4 – 2.2]	0.8 [0.4 –1.5]	0.9 [0.4 – 2.2]
Urban area (rural)	1.5 [0.7 – 3.0]	0.2 [0.4 – 1.2]	1.4 [0.7 – 2.7]
"Frequently" consult with colleagues ("very seldom" or "no")	0.4 [0.3 – 1.6]	1.0 [0.5 – 1.8]	0.8 [0.3 – 2.0]
"Have experience of consultation" with ethics committees (have no experience)	2.1 [0.8 – 6.2]	1.0 [0.4 – 2.2]	6.4 [2.5 – 16.3]

* Reference category in parentheses

* If the independent variables were missing, there were indicated as reference data.

The results showed that physician characteristics and employment characteristics were not significant explanatory factors for preferences pertaining to the withholding or withdrawal of treatment. Physicians possessing experience in consultation with ethics committees or medical ethicists were more likely to respond that the administration of enteric nutrition "Should be withdrawn" in Case 3 (OR, 6.4; 95% CI, 2.5–16.3; P < 0.001). Significant relationship to other factors was not observed in any case.

## Discussions

Our internet survey has several methodological problems. First, the publicity of the survey primarily targeted internist physicians in primary care settings. The distribution of the physician specialties shows that despite responses being obtained from a certain proportion of emergency care physicians and surgeons, the responses from internists comprised a large proportion. Therefore, the results of this study cannot be representative of the overall awareness of Japanese physicians on these issues. Additionally, this study was a web-based survey in which the responses were obtained from a homepage; this clearly indicates that the set of physicians accessing the homepage were not representative of the typical Japanese physician population [12]. In all likelihood, the physicians who participated in the survey were largely physicians with an interest in treatment decisions concerning "life-sustaining treatment"; thus, the sample analyzed must necessarily include substantial bias.

We received a number of major suggestions from this survey despite the above-noted limitations. In the course of routine treatment, most physicians had personal experience of having to make difficult decisions like those presented in the three scenarios. Nonetheless, we observed discrepancies in judgment among physicians related to specific "life-sustaining treatment" in specific scenarios. Further, we found disparities among physicians with regard to whether such medical interventions were "life-sustaining treatment." For example, there were great differences in the respective proportions of physicians who regarded nutritional supplementation by tube feeding and respiratory assistance by an artificial respirator as interventions that "should" be undertaken for patients with a prolonged disturbance of consciousness. In such circumstances, we found that physicians demonstrated a greater resistance to the attachment of an artificial respirator than to the initiation of tube feeding, and the extent of withholding of such treatment was actually greater. As has previously been studied, we hypothesized in similar fashion that for physicians, the attachment of a respirator was an alternative to which they exhibited a greater resistance among potentially permanent treatments; this is because of the high invasiveness of this procedure [13,14]. In our case scenarios, approximately half of the physicians surveyed responded that tube feeding "should be initiated" in situations of judgment during the acute phase of an illness where the potential for recovery remained; however, approximately the same number of physicians responded that medical treatment "should not be withdrawn" in scenarios where more than half a year had passed, and the medical potential for recovery was extremely low. This result underscores the strength of the resistance to the withdrawal of treatment relative to that for the withholding of treatment [15,16].

Despite the fact that the results for descriptive analyses were virtually the same with regard to the withholding of treatment in Case 1 and the withdrawal of treatment in Case 3, the lack of high concordance in these responses suggests that there is no fixed consensus among physicians concerning the withholding or withdrawal of treatment. A greater number of affirmative opinions were obtained from physicians who possessed an experience in ethical consultations for the withdrawal of treatment for patients in whom the potential for recovery was extremely low, and the next of kin had requested the withdrawal of treatment. In other words, more affirmative opinions were obtained in Case 3; moreover, according to the general ethical principles, among our three cases, this case is understood to be the one in which the selection of withdrawal would be most valid [17]. This result suggests that there is a need for ethical consultants, and that an experience in ethical consultation is effective for producing judgments of greater validity in end-of-life care. Simultaneously, the fact that no significant relationship was observed between judgment and preference with regard to either physician characteristics or hospital characteristics suggests that there is no decision model from which to undertake ethical instruction in the current physician environment [18].

The physicians participating in our internet survey took part voluntarily after encountering survey publicity, despite the lack of any financial incentive. We therefore hypothesized that this group of physicians had a higher awareness of ethical issues in medical treatment than our target population of Japanese physicians in general. However, even among this group, we found that there was negligible consultation with colleagues, conference studies, or other such activities addressing ethical issues. In particular, nearly all of the physicians had no experience of activities such as applications to specialists in clinical ethics or to ethics committees. This finding may suggest that the environment and culture that allows physicians to consult with other medical staff is currently limited.

Another finding from our research is the fact that there is more than a slight discrepancy between the "should/should not" awareness of treatment options and what is actually being done. In particular, in Case 3, very few physicians who believe that enteric nutrition "should not" be withdrawn actually withheld or withdrew medical intervention. This fact signifies that there is a high resistance to genuine action, which is distinct from the issue of whether the withdrawal of treatment is valid. Strong considerations include psychological resistance concerning the causation of death through intentional acts by the physician, and the contravention of legal norms [19,20]. The intent of the treatment providers was more distinct with respect to the withdrawal of treatment than to the withholding of treatment. Consequently, we believe that such intent resides in a perspective of physician responsibility, and that the psychological resistance engendered by responsibility creates a disconnect between judgment based on ethical validity and actual treatment decisions.

We believe that the current survey results point to a plan that should be undertaken to ensure that difficult decisions regarding life-support care in medical settings are made with greater validity. First, we discern a need for individuals or organizations to provide specific support for clinical decisions that encompass ethically complex elements. In practice, the accessibility of hospital ethics committees and clinical ethicists must also be enhanced. Currently, the matters considered by ethics review committees in Japan primarily concern research, and these bodies do not serve as organizations supporting clinical decision-making in actual clinics [21]. Additionally, while specialists in medical ethics exist, an extremely limited number of personnel actually travel to treatment settings and are able to establish close communication with treatment staff and address the resolution of clinical problem on-site. Infrastructural investments in personnel should be made.

Second, there is a need to reach a certain degree of consensus regarding the conduct of ethical decision-making in end-of-life care taking into account of the tendency Japanese physicians' attitudes toward some different clinical situations; variations of specific treatments; withholding or withdrawal of treatment. The term "life-sustaining treatment" has generally been perceived as a negative image of a practice not commonly done; however, our research suggests that there is a great discrepancy as to whether specific medical interventions based on detailed scenarios constitute "life-sustaining treatment" even among physicians. A more detailed study is required on specific medical interventions, rather than that on the image projected by "life-sustaining treatment." In Japan, in particular, notwithstanding the presence of major confusion in treatment settings, we are currently far from a consensus of opinion on the ethical differences and equivalencies in the withholding and withdrawal of medical interventions. The withholding of treatment that should not be carried out and the withdrawal that is judged to be valid must be deliberated from a greater number of bases and perspectives.

Finally, in clinical matters where the consideration of ethical issues is strongly indicated, we look forward to clinical conferences and other efforts toward regular and active information exchange among medical personnel.

## Conclusion

The study indicated that Japanese physicians tend to harbor greater negative attitudes toward the withdrawal of life-support care than its withholding. On the other hand, they favor withholding invasive life-sustaining treatments such as the attachment of a respirator over less invasive and long-term treatments such as tube feeding. Discrepancies were demonstrated between attitudes and actual behaviors. Physicians may need systematic support for appropriate decision-making for end-of-life care.

## Competing interests

The author(s) declare that they have no competing interests.

## Authors' contributions

SB: Designing overall research. Completing the survey and analysis

AA: Developing and modifying the survey questions

Sponsor's Role: National governmental funding agency

## Appendix

The case scenarios used in the survey

### Case 1

An 84-year-old man with mild dementia at the outset and Level 3 care requirement* for daily living, hospitalized for unilateral paralysis in conjunction with loss of consciousness due to left internal carotid artery embolism. Life was preserved in the acute phase, but the patient is wholly incapable of coherent conversation at 6 days after admission. The patient is completely bedridden and requires a change of position every few hours. There is pooling of saliva and sputum in the mouth, and oral suctioning is performed approximately 10 times per day. The administration of enteric nutritional agents as part of the nutritional management is required to maintain the nutritional status. When the administration of these nutritional agents via nasogastric intubation or the creation of a gastrostomy was explained to the family, their response was, "As long as he will not suffer, we will leave the decision to you." There is no information from which to infer the prior wishes of the patient.

*Level 3 care requirement means the requirement of support from others for daily bathing and toileting according to the category decided by the Ministry of Health, Labour and Welfare. They cannot stand up and walk by themselves.

Question 1: "What do you think should be done with regard to the initiation of enteric feeding for the aforementioned patient?"

Question 2: "What do you do with regard to the initiation of enteric nutrition for such a type of patient?"

### Case 2

An 84-year-old man hospitalized for unilateral paralysis in conjunction with loss of consciousness due to internal carotid artery embolism, with the clinical progress in the acute phase the same as in Case 1. Enteric nutrition was initiated by nasogastric intubation on day 6. The paralysis and state of consciousness remained unchanged, and the overall condition stabilized as bedridden, with regular administration of enteric nutrition alone apart from several drugs given. The respiratory status deteriorated abruptly on day 20 of admission, and major aspiration pneumonia was developed. Hypoxemia and labored breathing developed, and the attachment to an artificial respirator became necessary for life saving and recovery. Complete recovery from pneumonia may be possible, but attachment to the artificial respirator for several weeks is required, and depending on the circumstances, tracheotomy may be necessary. The family has again responded, "As long as he will not suffer, we will leave the decision to you."

Question 3: "What do you think should be done with regard to the attachment of an artificial respirator for such a type of patient?"

Question 4: "What do you do with regard to the attachment of an artificial respirator for such a type of patient?"

### Case 3

An 84-year-old man hospitalized for unilateral paralysis in conjunction with loss of consciousness due to internal carotid artery embolism, with the clinical progress in the acute phase the same as in Cases 1 and 2. Tube feeding was initiated by nasogastric intubation on day 6. The paralysis and state of consciousness remained unchanged, and the overall condition stabilized as bedridden. A gastrostomy was then performed, and the patient was transferred to a recuperative unit on day 28. After 6 months, the patient was bedridden, unable to communicate his will, and was in a state still requiring oral suctioning 10 times per day and changes of position several times a day. On a certain day, the routine visitors from the family (the wife and the oldest son) made a request to you as the ward physician: "We cannot go on seeing him suffer; we would like you to remove the feeding tube."

Question 5: "In circumstances similar to those described above, do you think that enteric nutrition should be withdrawn pursuant to a family request?"

Question 6: "What do you do regarding the withdrawal of artificial nutrition in cases such as the one described above?"

## Pre-publication history

The pre-publication history for this paper can be accessed here:


